# Supplementary figures and images for: Identifying the genes impacted by cell proliferation in proteomics and transcriptomics studies
Source: PLoS Comput Biol. 2022 Oct 6;18(10):e1010604. doi: 10.1371/journal.pcbi.1010604 (PMC9578628; doi:10.1371/journal.pcbi.1010604)

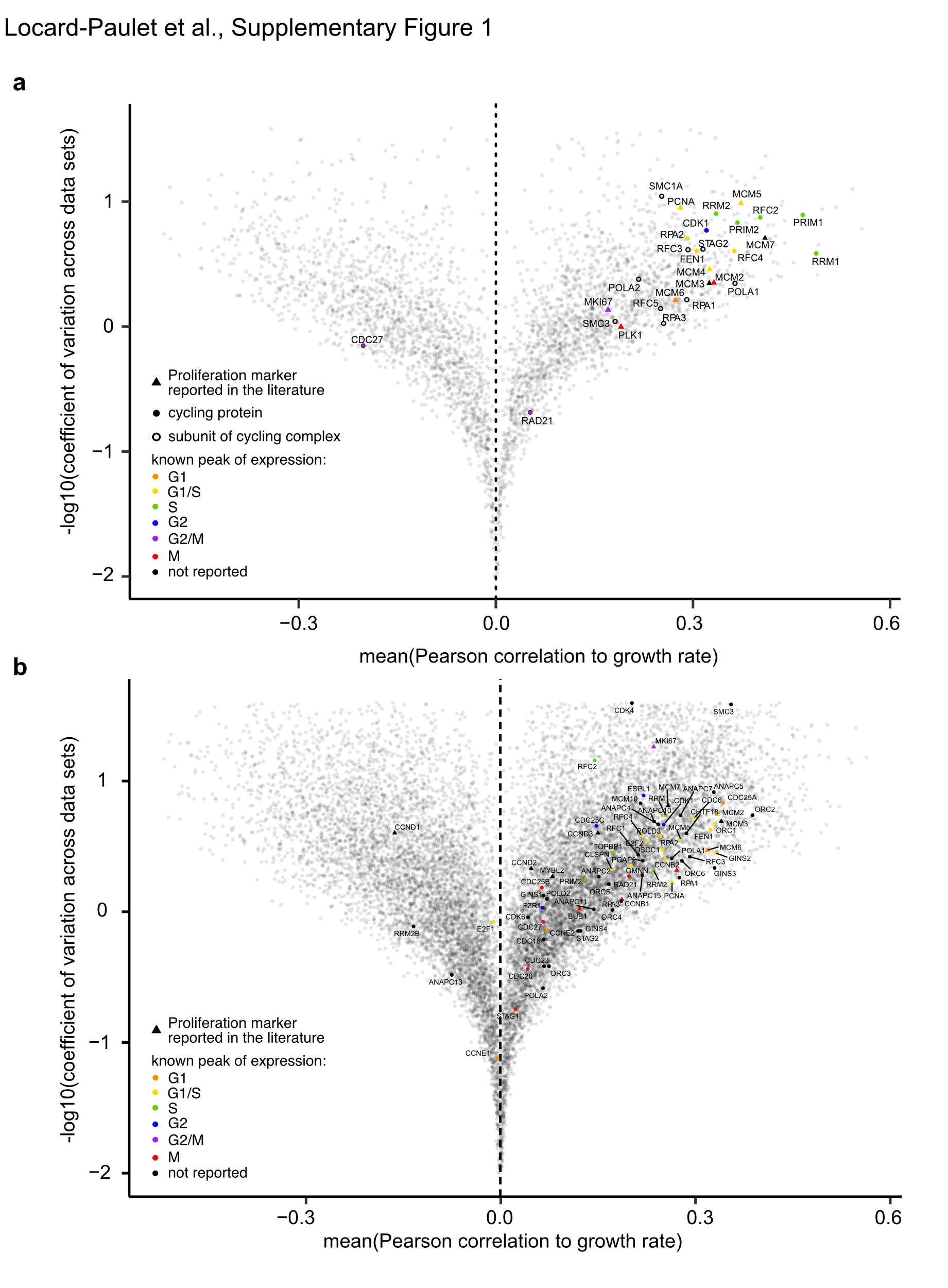

Supplement: S1 Fig — Volcano plots showing the mean correlation of proteins (a) or transcripts (b) to growth rates in the NCI60 data sets (horizontal axis) and the -log10(coefficient of variance) across all the data sets (vertical axis). Proteins quantified in less than 3 data sets were excluded in (a). Proteins/genes of interest are highlighted, and proliferation markers identified from literature search are indicated with triangles. These were color coded based of their expression peak according to Santos et al. [17]. (TIFF) [file pcbi.1010604.s001.tiff]

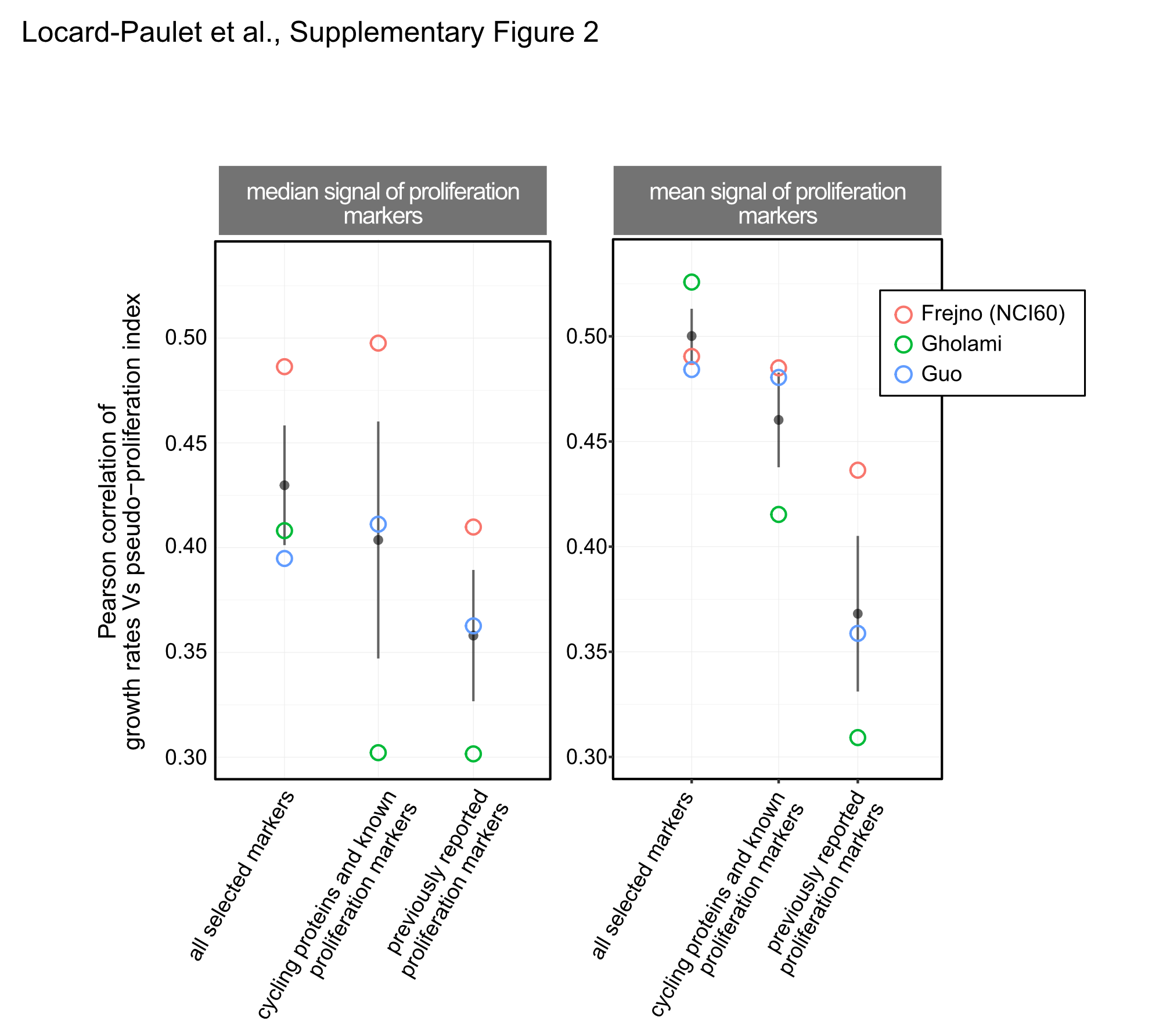

Supplement: S2 Fig — Pearson correlations between pseudo-proliferation index and growth rates in the proteomics data sets containing NCI60 cells presented in (Fig 1a) using the median (left panel) or mean (right panel) signal of the three sets of proliferation markers as selected in (Fig 1b) (grey area), all the previously reported proliferation markers, or the previously reported proliferation markers and cycling genes with the exclusion of RAD21. Grey points and bars are mean and confidence intervals across data sets. The right panel is the same as Fig 1c, it is reported here for direct comparison with the left panel. (TIFF) [file pcbi.1010604.s002.tiff]

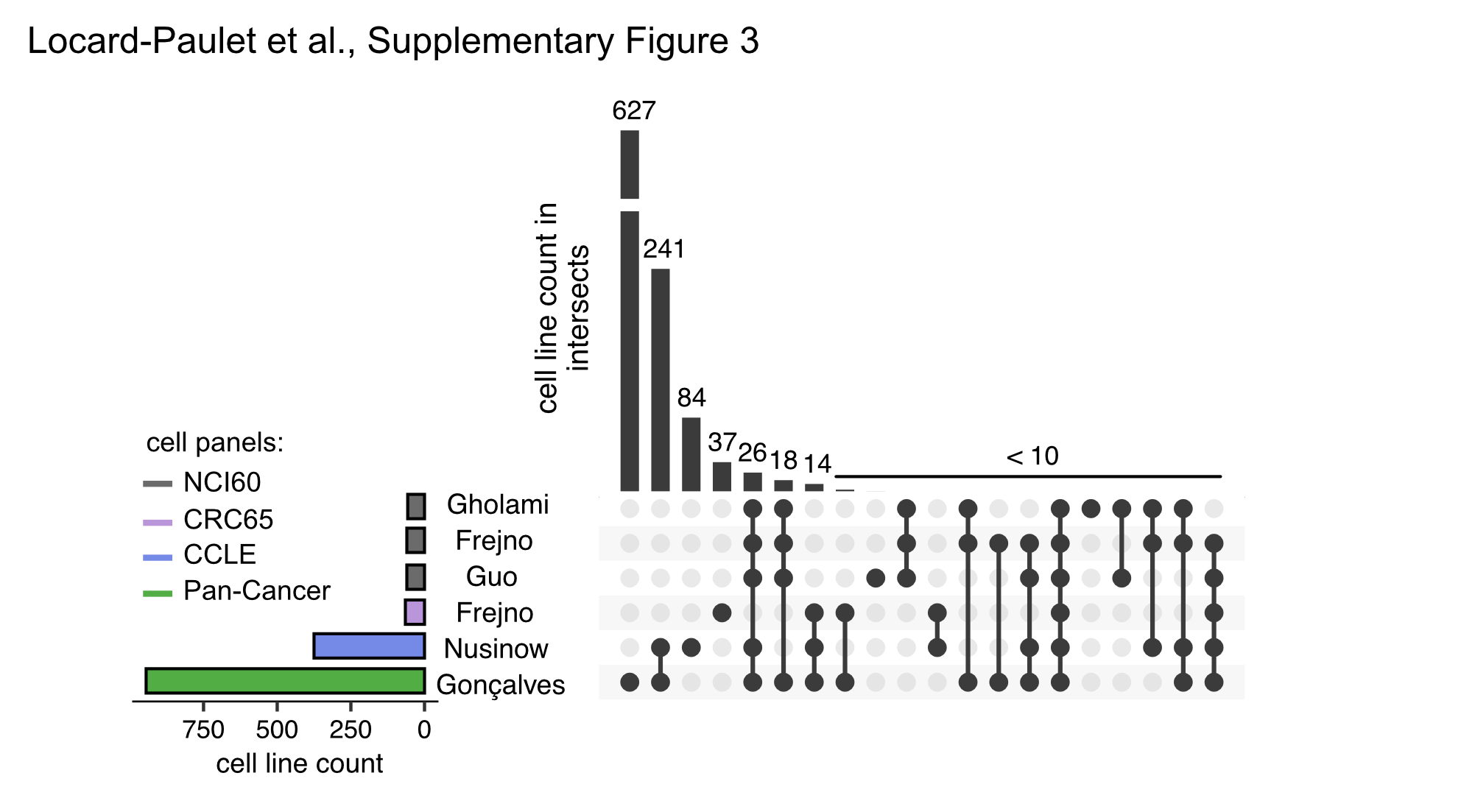

Supplement: S3 Fig — Number of cell lines in the proteomics data sets used in the study. The total number of cell lines in the data sets are indicated in the left-hand side bar plot (color-coded by the cell line panel). The cell lines present in multiple data sets are indicated by the bar plot on the top: number of protein groups detected in the data sets indicated by a dot on the dot plot. Each data set is identified by the first author’s name. (TIFF) [file pcbi.1010604.s003.tiff]

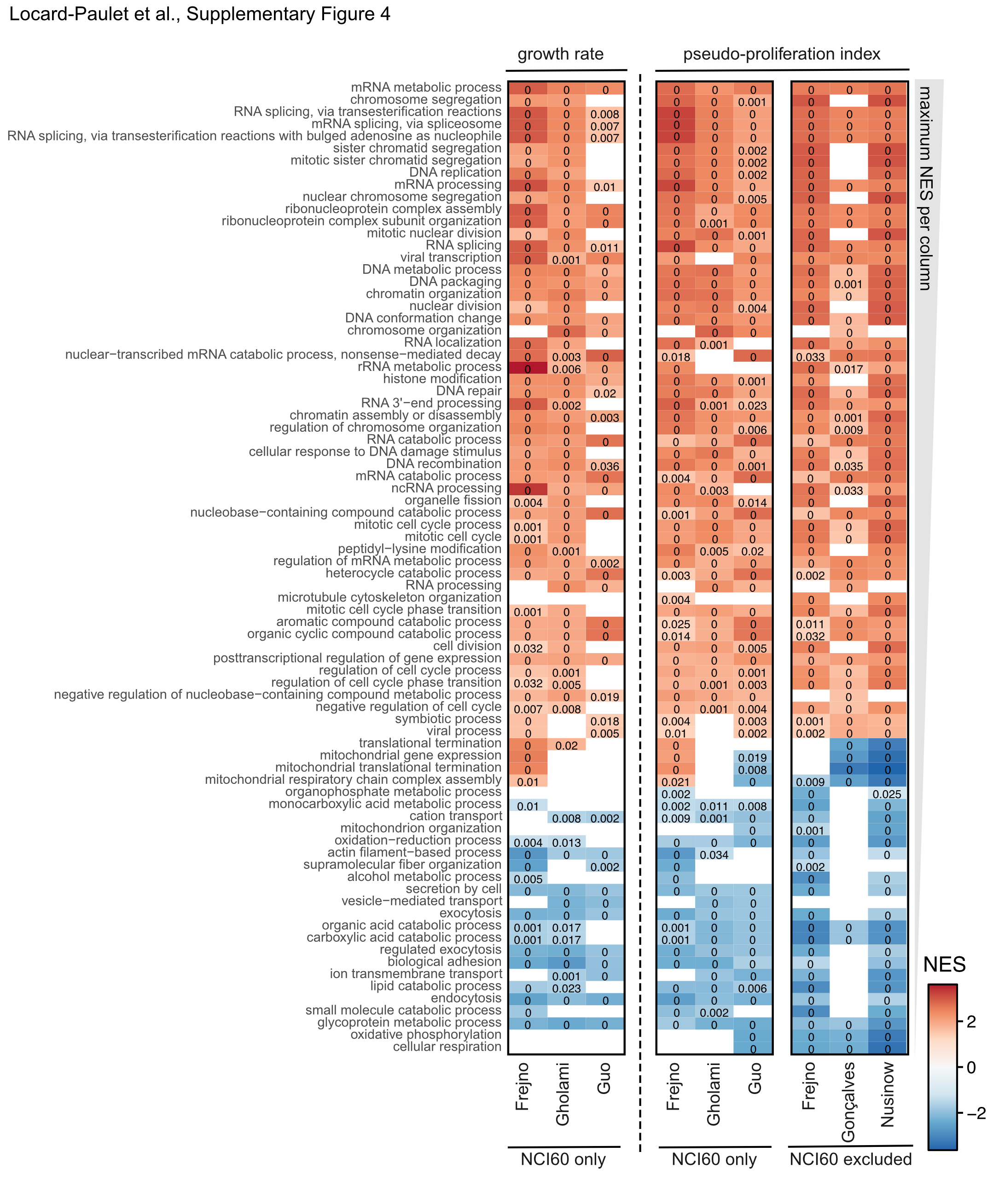

Supplement: S4 Fig — For each data set, genes (with the exception of the genes used for calculating pseudo-proliferation index) were ranked based on their correlation to growth rates or correlation to pseudo-proliferation index (left and right panel, respectively). Gene set enrichments were performed using the “gseGO” function from the R package clusterProfiler v 3.18.1, resulting p-values are indicated in each tile, as well as color-coded normalized enrichment scores (NES). Only the annotations from biological processes are included, they are ordered by decreasing maximum NES per data set (top 80 enriched GO terms, see material and methods for a detailed description of the procedure used to reduce GO redundancy). Data sets are labeled based on the first author’s name, enrichments were performed independently on the NCI60 cell lines or cell lines with no reported doubling time (“NCI60 only” and “NCI60 excluded”, respectively). (TIFF) [file pcbi.1010604.s004.tiff]

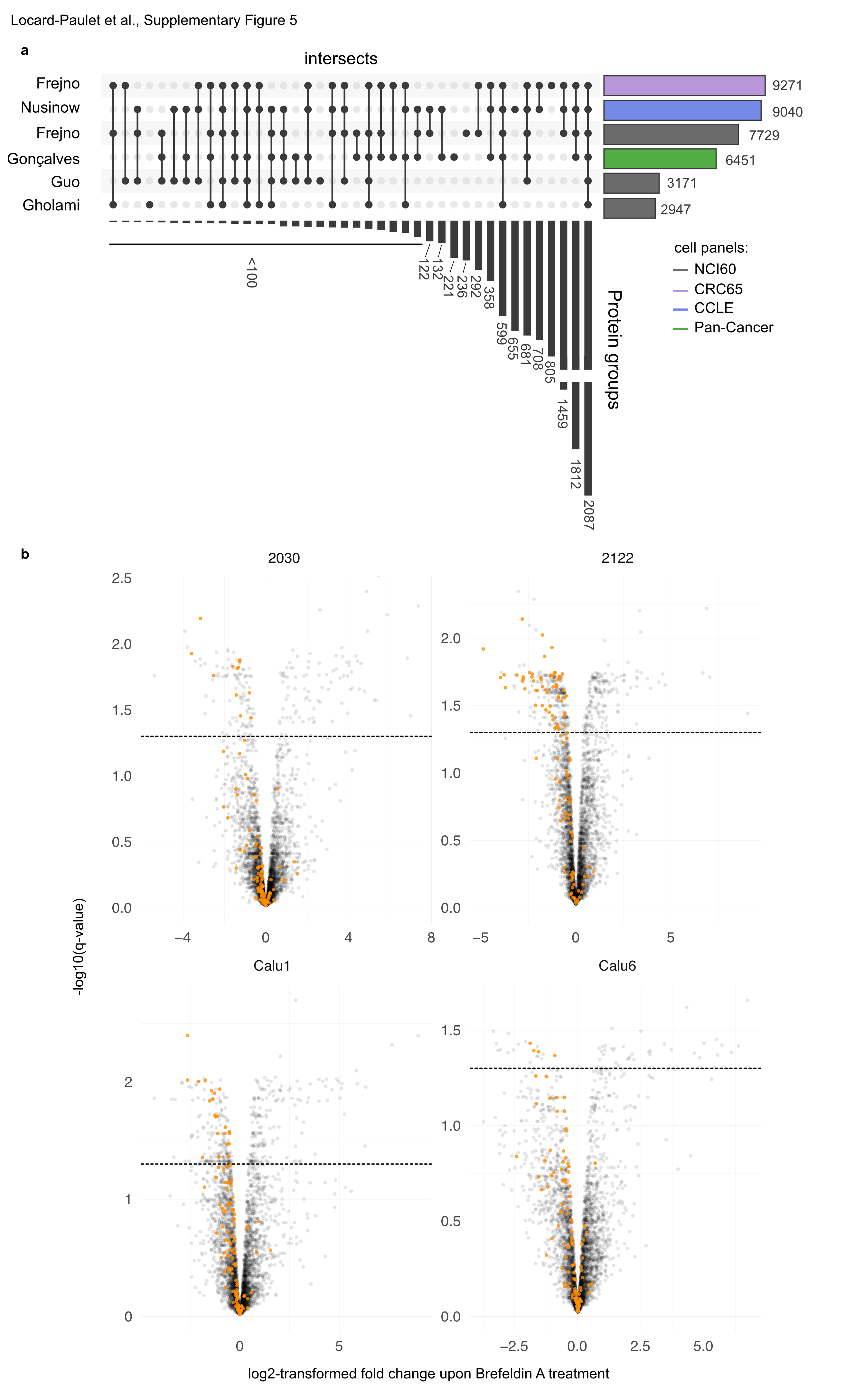

Supplement: S5 Fig — a) Protein coverage of the proteomics data sets used in the study (after isoform removal and accessions homogenization—see material and methods). These are identified by the first author’s name (left). The data set “Frejno” contained two independent MS searches of different cell line panels, we kept them separated. The total number of protein groups detected in the data sets are indicated in the bar plot on the right-hand side (color-coded by the cell line panel). The protein groups identified in multiple data sets are indicated by the bar plot on the bottom: number of protein groups detected in the data sets indicated by a dot on the dot plot. b) Volcano plots for each cell line treated with Brefeldin A. Genes constituting the proliferation signature are highlighted in orange. The dashed line corresponds to a q-value of 0.05. (TIFF) [file pcbi.1010604.s005.tiff]
