# Supplementary material for: Identifying the genes impacted by cell proliferation in proteomics and transcriptomics studies
Source: PLoS Comput Biol. 2022 Oct 6;18(10):e1010604. doi: 10.1371/journal.pcbi.1010604 (PMC9578628; doi:10.1371/journal.pcbi.1010604)
Supplement: Attachment — Submitted filename: ReviewersAnswer_Final.docx [file pcbi.1010604.s010.docx]

Dear editor,

We want to thank you and the reviewers for spending the time to review or manuscript. Their comments were very relevant and useful. Here are the major changes that we made to the previous version of the manuscript:

- We changed the “proliferation confounders” to “proliferation signature genes” throughout the manuscript.
- We spent more time to select the proliferation markers used to calculate pseudo-proliferation index (Figure 1). This resulted in an updated list of 25 genes.
- We added the data set from Gonçalves *et al.* to the proteomics data. It contains the proteomes of many cell lines, which makes it ideal for this study.
- In view of the new results, we decided to only keep one threshold to select the proliferation signature genes (Figure 2). In our opinion, this is simpler to communicate and to use than having low- and high-confidence signature genes. Furthermore, the thresholding curves that we used (Figure 2a-b) only presented one “clear” elbow to base our choice of thresholding value on.

Overall, these changes did not affect much the final set of proliferation signature genes that we present, while improving the manuscript. You can find below our point-by-point answer to the reviewers’ comments. We hope that they will be satisfied with the changes made to this manuscript.

Thank you again for your time.

On behalf of the authors,

Lars Juhl Jensen

Reviewer's Responses to Questions

**Comments to the Authors:
Please note here if the review is uploaded as an attachment.**

Reviewer #1: The manuscript by Locard-Paulet et al. describes a compilation and analysis of transcriptomic and proteomic datasets to identify genes whose expression is highly correlated with cell proliferation rates. The study is straightforward. The authors queried all the available datasets and identified gene expression signatures of cell proliferation with high confidence. In a clever extension, they also used these markers to predict proliferation rates in cases/cell lines for which cell proliferation rates are unknown. Overall, the work is solid, and the datasets will be of value to researchers. However, the way the authors pitch their findings is unfortunate and, in my opinion, wrong. I do not understand why the authors recklessly undermine the significance of their work. For the reasons I will detail below, I think the manuscript ought to be re-written before publication in any journal.

MAJOR CONCERNS
1. The authors present their findings throughout the manuscript as if the genes they identified are garbage and irrelevant. They call them confounders and envision their work analogous to the CRAPome reported previously for spurious protein-protein interactions. I cannot fathom the reasons for this genuinely bizarre interpretation. Growth rate as a variable is of immense significance in itself. Innumerable omic screens are done with cell proliferation rates as the critical phenotypic output (probably most cancer-related, cell-based screens). Their own 'use case 2' makes this point, where many of their 'confounders' are validated targets implicated in cancer. For example, the earlier work they cite from just one dataset (ref 1) identified the Myc/E2F network as a major contributor to proliferation rates, which is highly physiologically relevant. There is nothing 'confounding' or 'CRAPome’-related. The authors did some nice analyses, querying multiple datasets, and coming up with gene lists that will undoubtedly help others. They should present their work as follows: First, describe their goal exactly as it is: Identify gene expression signatures associated with proliferation rates. Whether such signatures are confounders or CRAPome-related depends on each screen's purpose. Then, explain that for cases where growth rate is the point of interest, users will have a gold standard reference at their disposal. If the growth rate is not of interest (similar to their 'use case 1') as in screens looking for a drug target, their list will help eliminate indirect effects due to growth rate changes. I understand that this would require a significant re-editing of the whole manuscript, but it would cast the work in a more positive, inclusive context.

We thank Reviewer #1 for his/her comment. We made changes throughout the manuscript to highlight the interest of the proliferation signature with a more positive light. In addition to rewriting specific parts, we replaced the “confounders” and “confounding genes” by “a gene signature for cell proliferation” (“proliferation signature” or “signature genes” depending on the sentence). We believe that it makes the manuscript more appealing.

We also added in the abstract how the proliferation signature can be used to explore phenotypes:
*“We show how to use this resource to help in interpretation of in vitro drug screens and tumor samples. It informs on differences of cell proliferation rates between conditions where such information is not directly available. The signature genes also highlight which hits in a screen may be due to proliferation changes; this can either contribute to biological interpretation or help to focus on experiment-specific regulation events otherwise buried in the statistical analysis."*

2. I find their development of the pseudo-proliferation index very useful. However, they offer no experimental validation. The authors should measure the doubling times of some cell lines for which no data is available and see if they match their computational estimates. This is a simple experiment that should be done to test their predictions.

While this is a computational biology study, we agree that an experimental validation to determine the accuracy of the pseudo-proliferation index would be interesting. However, according to our collaborators in mass spectrometry laboratories, it would not be easy to accurately measure cell growth rates that match the growth rates of the cells at the time right before mass spectrometry analysis. Cell doubling times can vary depending on how/when cells were split, and the exact medium composition at the time of lysis. Furthermore, cell proliferation can be inhibited by contact-initiated inhibition of proliferation before they are lysed. Finally, doubling times are usually not measured on the same batch as the one used for MS analysis.

Realizing these caveats of reported doubling times, in this new version of the manuscript we spent more time selecting the proliferation markers used for calculating the pseudo-proliferation index. To compare growth rates and pseudo-proliferation index, we only used the growth rates of the Developmental Therapeutics Program (DTP) and the data sets that either ordered cell pellets from there (Guo *et al.* and Gholami *et al.*) or received cells from the DTP and grew them according to the DTP recommendation (Frejno *et al.* and the two NCI60 transcriptomic data sets) (see new Figure 1). Nusinow *et al.* used different media for growing the NCI60 cell lines in their study, so we could not be sure that it did not affect relative cell growth. For this reason, we removed this data set from the analysis of cell growth rates.

We believe that the experimental validation that Reviewer #1 suggests would only be possible in a team that has expertise in studying cell cycle, works with tightly controlled cell growth conditions, and performs transcriptomics and/or MS-based proteomics. This would not only require a significant amount of work, but would also change the scope of the manuscript away from computational biology. Also, our focus is not the accurate estimation of cell growth rates but rather to identify a set of proteins that are involved in cell division and initiation of replication, the expression of which are likely impacted by growth rate. Being a computational group, and submitting this manuscript to a computational journal, we hope that the editor will understand our decision not to undergo experimental validation.

MINOR
Line 229: Small typo. Change Befeldin to B*r*efeldin.

This typo has been corrected.

Reviewer #2: Review of “Identifying the genes impacted by cell proliferation in proteomics and transcriptomics studies” by Locard-Paulet, Palasca and Juhl Jensen.

In the work presented in this manuscript, Locard-Paulet and co-authors revisit recently published transcriptomics and proteomics papers comparing large numbers of cell lines, looking across all cell lines and datasets for genes (or proteins) whose expression (or abundance) correlate with cell proliferation and therefore possibly confounding the interpretation of experiments whose design did not adequately take differential cell proliferation or proportion of cells at different cell cycle phases into account, for example when comparing treated cells with non-treated controls. Although many researchers are undoubtedly aware of this, and correctly identify such effects from their data (e.g. by looking at enriched biological processes corresponding to DNA replication or cell division - however incomplete the gene annotation), the current manuscript tells a compelling story that will serve to increase awareness of cell cycle confounders and provide a practical index based on a set of proteins strongly and consistently affected by changes in cell proliferation for estimating relative cell proliferation in proteomics datasets without information on cell growth rates. The authors also demonstrate how the index and confounders can be used in the context of cancer drug treatments, where the drug may directly affect the abundance of one or more of the confounders, showing that this small set of confounders can be used as a metaphorical lens through which one can look at different types of data.

The manuscript addresses an important problem and is very well written. The conclusions are supported by the data. All relevant details on the methods, underlying data and code are available as supplemental information and on Zenodo, enabling others to use and modify this pseudo-proliferation index or adapt it for other purposes. I could access these files and open the Cytoscape files without issues.

However, I do have only a few questions to the authors, and some minor comments and suggestions:

Robustness of the index:

How robust is the pseudo-proliferation index? It is calculated as a mean of the signal of 22 proteins, making it sensitive to an outlier that could be caused by a single false peptide identification or interfering species. Would it not be better to use the median, or some other more robust statistic?

We fully agree with Reviewer #2 that mean may not have been the best choice and thought that median could indeed be a more robust alternative. Thus, we followed this suggestion and calculated pseudo-proliferation index using the median of the new set of selected proliferation markers (see the answer to the second comment of Reviewer #1). We were surprised to see that using the median does not perform as well as using the mean. The Pearson correlation of pseudo-proliferation index with the growth rates of the NCI60 cell lines are lower, and the variance between proteomics data sets is higher. This is now shown in the new Supplementary Figure 2 of the manuscript. In light of these results, we decided to keep using the mean signal of the proliferation markers.

Will counting all MCM proteins individually not give too much weight to the MCM complex?

We understand this comment, and it is true that the signal of the MCM complex contributes largely to the pseudo-proliferation index. However:

- the proteins the most correlated with growth rates (*i.e.* the best candidates for reliable proliferation markers) are part of complexes (see figure 1b). Averaging their signal would give more weight to proteins that are not as good markers of cell proliferation.

- if we decided to average the signal of the MCM proteins, we would for consistency need to also average the signal of subunits of all other complexes too. Many of the proteins selected to calculate the pseudo-proliferation index are involved in complexes, and several of these complexes in turn form even larger complexes. Averaging over the subunits of each complex would in effect imply putting less weight on the subunits of large complexes than on other individual marker proteins. Moreover, since not all proliferation markers are quantified in every data set, “simply” averaging the signals of complex subunits would result in giving them different weights in different data sets. In our view, if one were to assign different weights to different markers, this should be done through developing an optimized scoring scheme, *e.g.*, by applying machine learning. However, for this to be a viable option, an independent test set with reliable doubling times would be essential. As discussed above, this is not something that we would be able to produce, and it would change the scope of the paper substantially.

For these reasons, we decided to use all independent marker signal for calculating the pseudo-proliferation index.

Applicability of the index:

The genes/proteins selected for the pseudo-proliferation index calculation include only those previously functionally annotated with “DNA replication”, and none of those annotated with “chromosome segregation” (though RAD21 and MCM4 are apparently known to peak in the M-phase). The genes/proteins in the index were those with the highest correlation with known growth rates in the data used. How representative is this set for all types of confounding issues related to cell proliferation, in other cell lines or model organisms? Perhaps the authors could comment on this in the discussion?

We did not use functional annotation as a criterion to select the proliferation markers used for pseudo-proliferation index calculation, although Supplementary Figure 1 shows that among cycling genes, the ones peaking at S and G1/S are the most correlated to NCI60 growth rates. In the new version of the manuscript, Figure 1b shows that the genes selected for pseudo-proliferation index calculation are indeed largely related to DNA replication, but it is not the case for all the selected proliferation markers: MCM4 is expressed in M phase, MKI67 is a known proliferation marker that peaks at the G2/M transition; PLK1 is known to play specific roles during M phase, and we also selected proteins from the cohesin complex (STAG2, SMC1A).

Since proteins expressed in S-phase seem to be better proliferation markers, and many proliferation markers used to calculate pseudo-proliferation index are involved in DNA replication, we could worry that the proliferation signature that we determined could be biased towards proteins involved in DNA replication. However, the network presented in Figure 3 shows that the proliferation signature is also composed of many genes that are involved in chromosome segregation, or M-phase processes. We discuss this in the new version of the manuscript, when describing Figure 3:
“*Although the selected set of proliferation markers used to calculate pseudo-proliferation index mainly consists of genes involved in DNA repair, many genes of the proliferation signature are involved in other parts of the cell division cycle. Figure 3 shows that many of them are involved in M-phase processes such as chromosome segregation. This indicates that our strategy for selecting signature genes was not biased towards S-phase functions but retrieved genes for which expression corelated with cell proliferation for reasons that are yet to determine.*“

We did not fully understand what Reviewer #2 means by “all types of confounding issues related to cell proliferation”. In this study, we focused on cell growth rates. We suspect that the proliferation signature genes will also correlate with other confounding factors that correlate with cell growth rates, but further validation is needed to discuss it fully.

Regarding applying our proliferation signature to other cell lines and non-human model organisms: we can only make statements about the cell lines that were studied in the data sets we worked with. However, seeing the number of data sets and cell lines integrated in this study, we believe that the signature genes will correlate with relative cell growth rates of human cell lines that were not included here. We cannot make claims about it working in other organisms and we do not want to speculate about it in the paper. We believe, because the proliferation signature is composed of widely conserved cell cycle-regulated genes, that it could be applied to other eukaryotes. However, this would need to be confirmed by the analysis of data sets of other organisms that would provide matching relative cell growth rates. We believe that this is outside the scope of this analysis, especially since the primary focus of our work is on identifying the proliferation signature genes and not to create a method for predicting growth rate.

Minor comments/suggestions:

The authors correlate gene and protein abundance with the inverse of the cell doubling times, and use both “growth rate” and “inverse doubling time” semi-interchangeably in the manuscript. Would it make it easier for the reader to simply use “growth rate” [ln(2)/doubling time] throughout the text and in the figures, rather than both “growth rate” and “1/doubling time”?. The change should not alter the results or conclusions in any way.

We thank Reviewer #2 for this comment. It makes a lot of sense, and we changed the manuscript accordingly.

Figure 1b: What is meant by “coefficient of variance in the proteomics”? There seems to be a word missing at the end of this sentence, e.g. “data”. What intervals do the three sizes or the markers represent?

We modified the legend (now “coefficient of variance (Proteomics)”), and we added the values for coefficient of variance.

Figure 1, 2, 4 and 5: There are strictly speaking no x- or y-axis defined in these plots, though most readers would understand “x-axis” to mean the horizontal axis (abscissa) and “y-axis” the vertical (ordinate) axis.

Reviewer #2 is right; we modified the manuscript accordingly.

Reviewer #3: Locard-Paulet et al. focus on the systematic identification and quantification of cell proliferation effect in high-throughput transcriptomic and proteomics studies and its role as potential confounder in differential analyses. The authors set out to explore this effect using large scale cohorts of cell lines and identified 223 genes that are strongly associated with proliferating rates, expanding existent resources. Several examples and two use cases using both tumor and drug perturbation studies are presented to support the role of cell proliferation as a major factor in some contexts.

This analysis is aimed at assisting large scale hypothesis exploration studies to account for the confounding effects of growth rates in the identification of spurious associations. The computational and statistical analyses are well executed and the manuscript is clearly written. In general, this is an important question, nonetheless cell proliferation does not seem to affect baseline transcriptomics and proteomics extensively, and drawing the line between confounder and a biologically relevant feature is sometimes difficult. Hence, the standard application of cell proliferation as a covariate in large-scale association studies should not be recommended without prior evaluation. In fact, as already explored in cancer cell lines studies, cell proliferation impact is more pronounced in short term perturbation studies, such as drug response assays, as the authors confirm in the first use case, where faster growing cells show on average stronger drug responses.

Main comments:

1. It would be important to complement these analyses with more up-to-date and systematic information of cancer cell lines proliferation rates. This can be found in these couple of publications PMID:31068703 (doubling times for 708 cell lines in Supplementary Table 1) and PMID:35839778 (growth rates for 938 cell lines in Supplementary Table 1).

We thank Reviewer #3 for this comment, and for providing these references.

- Gonçalves *et al.* (2022) provides proteomics data for more than 900 cell lines. These data were a great addition to the proteomics data sets already included in our previous version of the manuscript. We thus added this paper to the pipeline for selection of the genes that constitute our updated proliferation signature. It was also good to confirm that adding a new data set had a very limited impact on the final list of signature genes.

- Regarding doubling times provided in Gonçalves *et al.*: the authors provide a value for “growth” in the supplementary table S1. Unfortunately, we were not able to get enough information to use it in our new version of the manuscript. We are not sure that growth was measured on the cells that were subjected to proteomics analysis. Furthermore, it was not clear to us how the value was calculated or defined. Since some of the values are negative, we conclude that it cannot be the growth rate per se. In the paper, the authors describe it as follows:
*“Cell line growth rates were represented as the ratio between the mean of the untreated negative controls measured at day one (time of drug treatment) and the mean of the dimethyl sulfoxide (DMSO) treated negative controls at day four (72 h post drug treatment).”*
We contacted the last author to get more information. He directed us to another corresponding author that we contacted twice without getting an answer. For these reasons, we did not include these growth measurements in our new version of the manuscript.

2. Some general statistics about the preponderance of cell proliferation could be informative to assess the importance of this in the datasets at hand. For example, using Principal Component Analysis (PCA) is the proliferation gene set associated with any principal component in specific? If so, how much variability does it explain?

The effect of cell proliferation could be spread across several principal components of a PCA, and it would be difficult to quantify how much variability it explains. To answer Reviewer #3’s question, we looked at the proportion of proteins that were considered significantly regulated in Ruprecht *et al.* and were signature genes. We believe that this shows how proliferation can confound such an experiment. We added this information in the manuscript for the use case with Brefeldin A and with docetaxel.

3. Page 9, line 195: “This indicates post-translational adjustment of protein quantities.”. This difference could also be attributed to post-transcriptional regulatory processes, correct?

We agree with Reviewer #3 and modified this sentence accordingly: “*This indicates post-translational adjustment of protein quantities and/or post-transcriptional regulatory processes.*”

Minor comment:

1. Page 3, line 82: “sixteen were quantified in minimum three of the NCI60 (...)”. Perhaps, “(...) in at least three (...)”?

We modified the manuscript accordingly.
